# Association of Inclusion of Medicare Advantage Patients in Hospitals’ Risk-Standardized Readmission Rates, Performance, and Penalty Status

**DOI:** 10.1001/jamanetworkopen.2020.37320

**Published:** 2021-02-17

**Authors:** Orestis A. Panagiotou, Kirsten R. Voorhies, Laura M. Keohane, Daeho Kim, Deepak Adhikari, Amit Kumar, Maricruz Rivera-Hernandez, Momotazur Rahman, Pedro Gozalo, Roee Gutman, Vincent Mor, Amal N. Trivedi

**Affiliations:** 1 Department of Health Services, Policy and Practice, Brown University School of Public Health, Providence, Rhode Island; 2 Center for Gerontology and Healthcare Research, Brown University School of Public Health, Providence, Rhode Island; 3 Providence Veterans Administration Medical Center, Providence, Rhode Island; 4 Department of Health Policy, Vanderbilt University School of Medicine, Nashville, Tennessee; 5 Northern Arizona University College of Health & Human Services, Flagstaff; 6 Department of Biostatistics, Brown University School of Public Health, Providence, Rhode Island; 7 Center for Statistical Sciences, Brown University School of Public Health, Providence, Rhode Island

## Abstract

**Importance:**

The Hospital Readmissions Reduction Program publicly reports and financially penalizes hospitals according to 30-day risk-standardized readmission rates (RSRRs) exclusively among traditional Medicare (TM) beneficiaries but not persons with Medicare Advantage (MA) coverage. Exclusively reporting readmission rates for the TM population may not accurately reflect hospitals’ readmission rates for older adults.

**Objective:**

To examine how inclusion of MA patients in hospitals’ performance is associated with readmission measures and eligibility for financial penalties.

**Design, Setting, and Participants:**

This is a retrospective cohort study linking the Medicare Provider Analysis and Review file with the Healthcare Effectiveness Data and Information Set at 4070 US acute care hospitals admitting both TM and MA patients. Participants included patients admitted and discharged alive with a diagnosis of acute myocardial infarction (AMI), congestive heart failure (CHF), or pneumonia between 2011 and 2015. Data analyses were conducted between April 1, 2018, and November 20, 2020.

**Exposures:**

Admission to an acute care hospital.

**Main Outcomes and Measures:**

The outcome was readmission for any reason occurring within 30 days after discharge. Each hospital’s 30-day RSRR was computed on the basis of TM, MA, and all patients and estimated changes in hospitals’ performance and eligibility for financial penalties after including MA beneficiaries for calculating 30-day RSRRs.

**Results:**

There were 748 033 TM patients (mean [SD] age, 76.8 [83] years; 360 692 [48.2%] women) and 295 928 MA patients (mean [SD] age, 77.5 [7.9] years; 137 422 [46.4%] women) hospitalized and discharged alive for AMI; 1 327 551 TM patients (mean [SD] age, 81 [8.3] years; 735 855 [55.4%] women) and 457 341 MA patients (mean [SD] age, 79.8 [8.1] years; 243 503 [53.2%] women) for CHF; and 2 017 020 TM patients (mean [SD] age, 80.7 [8.5] years; 1 097 151 [54.4%] women) and 610 790 MA patients (mean [SD] age, 79.6 [8.2] years; 321 350 [52.6%] women) for pneumonia. The 30-day RSRRs for TM and MA patients were correlated (correlation coefficients, 0.31 for AMI, 0.40 for CHF, and 0.41 for pneumonia) and the TM-based RSRR systematically underestimated the RSRR for all Medicare patients for each condition. Of the 2820 hospitals with 25 or more admissions for at least 1 of the outcomes of AMI, CHF, and pneumonia, 635 (23%) had a change in their penalty status for at least 1 of these conditions after including MA data. Changes in hospital performance and penalty status with the inclusion of MA patients were greater for hospitals in the highest quartile of MA admissions.

**Conclusions and Relevance:**

In this cohort study, the inclusion of data from MA patients changed the penalty status of a substantial fraction of US hospitals for at least 1 of 3 reported conditions. This suggests that policy makers should consider including all hospital patients, regardless of insurance status, when assessing hospital quality measures.

## Key Points

**Question:**

Is inclusion of Medicare Advantage patients in hospitals’ 30-day risk-standardized readmission rates associated with changes in hospital performance measures and eligibility for financial penalties?

**Findings:**

In this cohort study of 4070 US acute care hospitals, inclusion of data from Medicare Advantage patients was associated with changes in the performance and penalty status of a substantial fraction of US hospitals for at least 1 of the outcomes of acute myocardial infarction, congestive heart failure, or pneumonia.

**Meaning:**

These findings suggest that there is a need for the Centers for Medicare & Medicaid Services and other policy makers to consider incorporating outcomes of all hospitalized patients, regardless of insurance coverage source, in assessments of hospital quality.

## Teaser

This cohort study investigates the association of including Medicare Advantage patients in hospital risk-standardized readmission rates with hospital readmission performance metrics and incursion of financial penalties under the Hospital Readmissions Reduction Program.

## Introduction

Hospital readmissions are common, costly, and associated with substantial morbidity and mortality.^1^ Reducing readmissions has become a major priority for policy makers. Under the Hospital Readmissions Reduction Program (HRRP), the Centers for Medicare & Medicaid Services (CMS) has publicly reported qualitative assessments of hospital performance since 2009, including whether a hospital’s risk-standardized readmission rate (RSRR) is different from the national average readmission rate without identifying the precise RSRR estimate. Starting in 2012, CMS imposed financial penalties for hospitals with higher than expected rates of readmission.^2^ The HRRP calculates readmission rates exclusively for traditional Medicare (TM) beneficiaries, while rates of readmission for persons covered by the rapidly growing Medicare Advantage (MA) program are neither publicly reported nor financially penalized. The exclusion of MA patients from the HRRP has substantial implications because approximately 22 million of approximately 60 million Medicare beneficiaries (35%) were enrolled in MA plans in 2019.^3^

MA plans are private insurance plans that contract with Medicare, and they receive prospective capitated payments to bear the risk of financing covered services. They are, therefore, strongly incentivized to reduce costs of care and hospital readmissions. Hospitals’ readmission rates for the subset of Medicare beneficiaries with traditional coverage may not correlate with performance for all admitted patients,^4^ or even readmission rates for older adults with MA coverage. If this is true, then exclusively reporting readmission rates for the TM population may not accurately reflect hospitals’ readmission rates for older adults, or may incentivize hospitals to tailor readmission reduction efforts to patients with TM coverage. Conversely, if readmission rates for TM and MA patients are correlated, this finding would lend support to CMS’s current practice of reporting readmission rates exclusively for the TM population.

Here, we examined the implications of including MA patients on hospitals’ performance on readmission measures and eligibility for financial penalties. The study focused on readmission rates in acute myocardial infarction (AMI), congestive heart failure (CHF), and pneumonia, the first 3 targeted conditions included in the HRRP.

## Methods

### Data Sources

The cohort study was approved by the Brown University institutional review board, which waived the need for informed consent because the study was a minimal risk for participants and had no direct access to or contact with any individuals in the study. This study followed the Strengthening the Reporting of Observational Studies in Epidemiology (STROBE) reporting guideline.^5^

We linked claims data from the Medicare Provider and Analysis Review (MedPAR) files with the Healthcare Effectiveness Data and Information Set (HEDIS) from 2011 to 2015.^6,7^ In MedPAR Files, data on MA patients must be submitted by hospitals that receive disproportionate-share hospital payments or medical education payments from Medicare, although other hospitals may also submit data on MA patients. Hospitals that submit MA claims accounted for 92% of Medicare discharges between 2011 and 2013.^8^ Because hospitalizations occur in hospitals that do not report MedPAR data for MA enrollees, we linked the data to HEDIS records, which include data on index admissions (including admission and discharge dates) and an indicator of whether the patient was admitted within 30 days of discharge; this linkage increases sensitivity of identifying readmissions for all MA beneficiaries.^9^ With the exception of private fee-for-service plans and those with fewer than 1000 enrollees, all MA plans must report HEDIS data to CMS. The Medicare Beneficiary Summary File provided demographic beneficiary characteristics and TM vs MA enrollment status. We included MA plans regardless of whether they were owned by a hospital.

### Study Population

We applied CMS’s criteria to identify eligible TM and MA beneficiaries aged 65 years or older who were hospitalized in and discharged alive from nonfederal, short-term, acute care, noncritical access hospitals in the continental US and the District of Columbia with a principal diagnosis of AMI, CHF, or pneumonia between January 1, 2011, and November 30, 2015.^10,11,12,13^ Index hospital admissions for both TM and MA patients were identified from MedPAR as in previous work.^9^ We included only hospitals that admitted both MA and TM patients. MA status was determined according to MA enrollment as of the month of admission.

### Outcome

For both TM and MA beneficiaries, the outcome was readmission for any reason occurring within 30 days after discharge.^9^ Readmissions for TM patients were identified from MedPAR, which is designed to capture all inpatient claims in TM. For MA patients, we used both MedPAR and HEDIS; when a discordance between HEDIS and MedPAR records existed for MA patients, we included the readmission outcome as reported in HEDIS.^9^ This allowed us to capture readmissions for MA enrollees readmitted to hospitals that are not required to report data for MA enrollees but who were included in the HEDIS measures.

### Covariates

Covariates included age, sex, condition-specific comorbid conditions (consistent with CMS’s methods; eTable 1 in the Supplement), and year of hospital admission. Similar to CMS’s approach, we used principal and secondary diagnosis codes from the index hospitalization and hospitalizations in the 12 months preceding the index hospitalization to define the presence of comorbid conditions.^11,12,13^ Because outpatient claims are unavailable for MA enrollees, we used MedPAR data exclusively; of note, the inclusion of comorbidities from outpatient data do not result in meaningful changes in hospitals’ RSRRs.^14,15^

### Statistical Analysis

We applied the CMS’s methods used in the HRRP and separately calculated hospital-specific RSRRs for AMI, CHF, and pneumonia for TM patients only, for MA patients only, and for both TM and MA patients. Because CMS’s risk model was developed for TM patients, we assessed its performance by estimating its coefficients in TM patients, MA patients, and both MA and TM patients. We used the C statistic (area under the receiver operating characteristic curve) to assess the model’s performance in each population.^16,17^ We compared the coefficients’ estimates for the risk models when they were applied to either TM, MA, or the combined TM and MA populations for each condition.

Each hospital’s condition-specific 30-day RSRR for each risk model coefficients’ estimates was estimated as the ratio of its estimated and expected readmissions standardized to the national observed readmission rate for that condition and population (TM, MA, both); 95% CIs were computed using the bootstrap procedure.^18^ We calculated the estimated readmission risk using a multilevel logistic regression model with specific intercept for each hospital variable and linear additive adjustments for the covariates described above.^10,11,12,19^ We calculated the expected readmission risk similarly but relied on the average (mean) of the hospital-specific intercepts. eAppendix in the Supplement includes detailed information on these models. We used the Pearson coefficient, *r*, to assess the correlations in the 30-day RSRRs estimated using TM beneficiaries only, MA beneficiaries only, and all Medicare beneficiaries.

We ranked hospitals according to their 30-day RSRRs estimated for their TM patients only, MA patients only, and both MA and TM patients. We categorized hospital performance as better-than-expected (if a hospital’s RSRR is less than the national mean and the 95% CIs do not include the national mean), as-expected (if the 95% CIs around the RSRR include the national mean), and worse-than-expected (if a hospital’s RSRR is greater than the national mean and the 95% CIs do not include the mean) per the CMS’s measurement methods.^20,21^

We computed the excess readmission rate (ERR), which CMS uses to determine hospitals’ financial penalties.^20,21,22^ The ERR is the ratio between the estimated and expected readmissions. A hospital with an ERR greater than 1.0 means that its RSRR is higher than the national readmission rate. Hospitals with ERR greater than 1.0 and with 25 or more eligible discharges are subject to financial penalties. We constructed Bland-Altman plots,^23,24^ which show how the within-hospital differences in 30-day RSRRs between TM patients and both TM and MA patients vary across the within-hospital means of the 30-day RSRR for TM and both TM and MA patients.

For sensitivity analyses, we first estimated each hospital’s RSSR for both its TM and MA beneficiaries using the hierarchical modeling approach previously described except that for each variable we applied the regression coefficient estimated in the TM population. This approach allows us to assess whether any changes to the RSSR estimates with the addition of MA patients reflect underlying differences in the relative importance of risk factors for readmissions or changes in the case-mix of the hospital’s population. Second, we assessed whether hospitals’ shifts in performance reflect shrinkage to the national mean, which is stronger with a larger study sample that weights the global mean more, and could pull small hospitals with volatile ERRs toward the as-expected category. To do so, we sampled from the 2 populations (MA and TM) proportional to their rate such that the overall number of individuals in each hospital is the same as the size of the TM population; this approach allows us to compare whether it is the size of the sample or the addition of MA patients that is associated with the changes in performance. Last, we examined whether changes in a hospital’s performance status vary according to the fraction of MA beneficiaries at a given hospital for each condition.

Analyses were conducted with SAS statistical software version 9.4 (SAS Institute) and R statistical software version 3.6 (R Project for Statistical Computing). All statistical tests are 2-sided at α = .05. Data analyses were conducted between April 1, 2018, and November 20, 2020.

## Results

As shown in eTable 2 in the Supplement, between 2011 and 2015, there were 748 033 TM patients (mean [SD] age, 76.8 [83] years; 360 692 [48.2%] women) and 295 928 MA patients (mean [SD] age, 77.5 [7.9] years; 137 422 [46.4%] women) hospitalized and discharged alive for AMI; 1 327 551 TM patients (mean [SD] age, 81 [8.3] years; 735 855 [55.4%] women) and 457 341 MA patients (mean [SD] age, 79.8 [8.1] years; 243 503 [53.2%] women) hospitalized for CHF; and 2 017 020 TM patients (mean [SD] age, 80.7 [8.5] years; 1 097 151 [54.4%] women) and 610 790 MA patients (mean [SD] age, 79.6 [8.2] years; 321 350 [52.6%] women) hospitalized for pneumonia. A total of 4070 hospitals (eFigure 1 in the Supplement) discharged at least 1 TM and 1 MA patient with AMI (3167 hospitals), CHF (3838 hospitals), or pneumonia (4010 hospitals). Table 1 shows the number of discharges and 30-day RSRRs for TM, MA, and all Medicare patients for each condition. Demographic and clinical characteristics are shown in eTable 1 and eTable 2 in the Supplement.

**Table 1.** Readmissions After AMI, CHF, and Pneumonia for Traditional Medicare and Medicare Advantage Beneficiaries Between 2011 and 2015

Variable	No.		30-d RSRR, mean (SD), %
	Admissions	Hospitals^a^	
All traditional Medicare admissions			
AMI	812 656	3167	16.9 (1.1)
CHF	1 899 272	3838	21.7 (1.7)
Pneumonia	2 499 778	4010	16.4 (1.8)
All Medicare Advantage admissions			
AMI	318 850	3167	16.4 (1.0)
CHF	633 563	3838	21.4 (1.2)
Pneumonia	716 585	4010	16.3 (1.0)
All Medicare admissions			
AMI	1 131 506	3167	16.8 (1.2)
CHF	2 532 835	3838	21.6 (1.8)
Pneumonia	3 216 363	4010	16.4 (1.9)

Abbreviations: AMI, acute myocardial infarction; CHF, congestive heart failure; RSRR, risk standardized readmission rate.

^a^ Hospitals included those that admitted at least 1 traditional Medicare patient and at least 1 Medicare patient with the specified condition from 2011 to 2015.

### Assessment of Model Performance

Models estimating 30-day readmission risk after AMI, CHF, and pneumonia had modest discrimination and low calibration for both TM and MA patients (eFigure 2 in the Supplement). For each condition, model coefficients were largely similar across TM, MA, and all Medicare beneficiaries (eTable 3, eTable 4, and eTable 5 in the Supplement).

### Hospital-Specific RSRR

**Figure 1.**
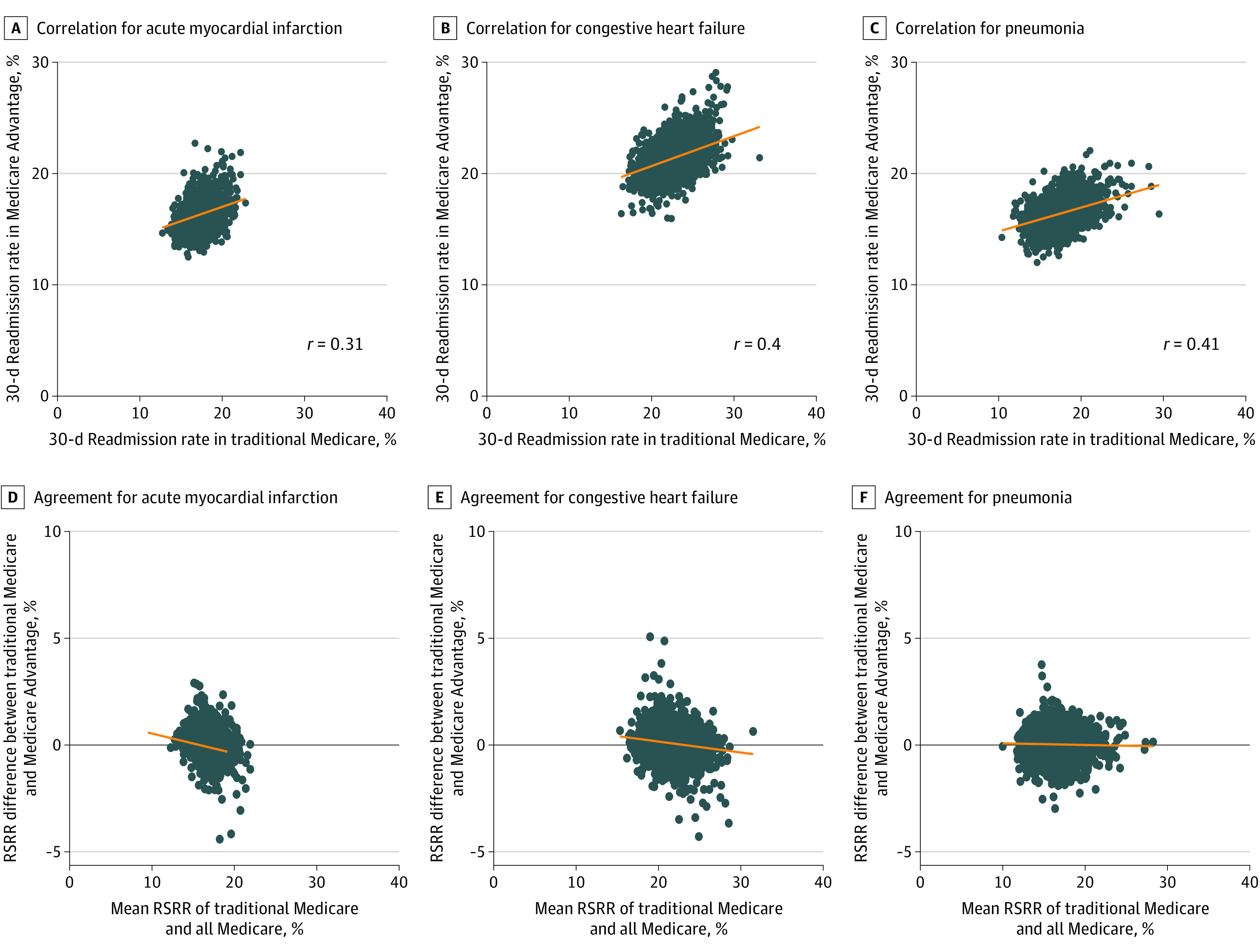
Comparison of 30-Day Risk-Standardized Readmission Rates (RSRRs) in Traditional Medicare With Medicare Advantage and All Medicare Patients Graphs show the correlation between 30-day RSRR for Traditional Medicare and Medicare Advantage patients for acute myocardial infarction (A), congestive heart failure (B), and pneumonia (C) and the agreement between 30-day RSRR for Traditional Medicare and all Medicare patients for acute myocardial infarction (D), congestive heart failure (E), and pneumonia (F). Orange lines were mathematically generated and fitted to the data to summarize the relationship among the variables in the x- and y-axes.

The mean hospital-specific 30-day RSRRs for TM, MA, and all patients are shown in Table 1 and in eFigure 3, eFigure 4, and eFigure 5 in the Supplement. There was correlation (*r* = 0.31 for AMI, *r* = 0.40 for CHF, and *r* = 0.41 for pneumonia) in estimated RSRRs between only TM and only MA patients (Figure 1), and correlation (*r* = 0.91 for AMI, *r* = 0.95 for CHF, and *r* = 0.97 for pneumonia) in estimated RSRRs between only TM and all Medicare patients (eFigure 6 in the Supplement).

Figure 1 also shows the Bland-Altman plots of agreement between RSRR for TM patients and for all Medicare patients. The slopes of the fitted lines were negative for AMI (slope = –0.09), CHF (slope = –0.05), and pneumonia (slope = –0.01), which means that for hospitals with higher RSRRs based on all Medicare patients, their respective TM-based RSRR is smaller than and underestimates the RSRR for both TM and MA patients combined. Differences in RSRRs were greater than 1.96 SDs for 178 hospitals (6%) for AMI, 211 hospitals (5%) for CHF, and 238 hospitals (6%) for pneumonia (eFigure 7 in the Supplement).

### Comparison of Readmission Rates for All Medicare Patients vs TM Patients Alone

On the basis of TM patients alone, the numbers of hospitals with better-than-expected or worse-than-expected performance for AMI, CHF, and pneumonia were 116, 421, and 589, respectively. For both TM and MA enrollees, 9 (8%), 36 (9%), and 37 (6%) of those hospitals changed their status for AMI, CHF, and pneumonia, respectively (Table 2). There were also 3051, 3417, and 3421 hospitals with as-expected performance for AMI, CHF, and pneumonia, respectively, for TM patients alone; of those, 90 (3%), 204 (6%), and 241 (7%) hospitals, respectively, became outliers according to both TM and MA patients (Table 2). Using TM data alone, we identified 68, 242, and 370 hospitals with worse-than-expected readmission rates in AMI, CHF, and pneumonia, respectively; of these low-performing hospitals, 7 (10%), 16 (7%), and 15 (4%), respectively, were no longer worse-than-expected according to both TM and MA patients (Table 2).

**Table 2.** Agreement in Hospital Rankings in 30-Day RSRR for TM and All Enrollees (TM and MA)^a^

Hospital 30-d RSRR in TM	Hospitals, No.	Hospital 30-d RSRR in MA and TM		
		Worse than expected	As expected	Better than expected
AMI				
No.		108	2970	89
Worse than expected	68	61	7	0
As expected	3051	47	2961	43
Better than expected	48	0	2	46
CHF				
No.		340	3249	249
Worse than expected	242	226	16	0
As expected	3417	114	3213	90
Better than expected	179	0	20	159
Pneumonia				
No.		478	3217	315
Worse than expected	370	355	15	0
As expected	3421	123	3180	118
Better than expected	219	0	22	197

Abbreviations: AMI, acute myocardial infarction; CHF, congestive heart failure; MA, Medicare Advantage; RSRR, risk standardized readmission rates; TM, Traditional Medicare.

^a^ RSRRs were derived from model (coefficients) fit using all hospitals with 1 or more admissions for both fee-for-service and MA.

There were 4021 hospitals with as-expected performance for at least 1 condition for TM patients alone, and of those, 470 (12%) became outliers based on both TM and MA patients (eFigure 8 in the Supplement). Another 826 hospitals had better-than-expected or worse-than-expected performance, and 81 (10%) changed their status after the inclusion of MA data.

Using model estimates based only on TM patients to estimate the hospital’s RSRR for the entire TM and MA population, we found that after inclusion of MA patients, 27 (1%) of the 4021 hospitals with as-expected performance for at least one condition and 51 (6%) of the 821 hospitals with better-than-expected or worse-than-expected performance for at least 1 condition changed their status. Condition-specific results are shown in eTable 6 in the Supplement.

eTable 7 in the Supplement shows the numbers of hospitals that changed outlier status when MA and TM beneficiaries were sampled such that the overall number of individuals in each hospital is the same as the size of the TM population. In total, 283 (7%) of the 4038 hospitals with as-expected performance for at least 1 condition and 100 (14%) of the 738 hospitals with better-than-expected or worse-than-expected performance for at least 1 condition changed their status. Of 445 hospitals with worse than expected readmission rates for at least 1 condition according to TM data alone, 55 (12%) were no longer worse than expected after the inclusion of MA data.

eTable 8, eTable 9, and eTable 10 in the Supplement show the number of hospitals that changed outlier status according to their fraction of MA admissions for each condition. A total of 247 hospitals (21%) in the upper quartile, 154 hospitals (10%) in the second quartile, 96 hospitals (6%) in the third quartile, and 54 hospitals (3%) in the lowest quartile changed status for at least 1 condition. In each quartile, 14 (10%), 8 (5%), 14 (10%), and 2 (3%) hospitals, respectively, changed status from worse-than-expected (higher RSSR) to as-expected or better-than-expected (lower RSRR).

### Changes in Penalty Status After Including MA Patients

**Figure 2.**
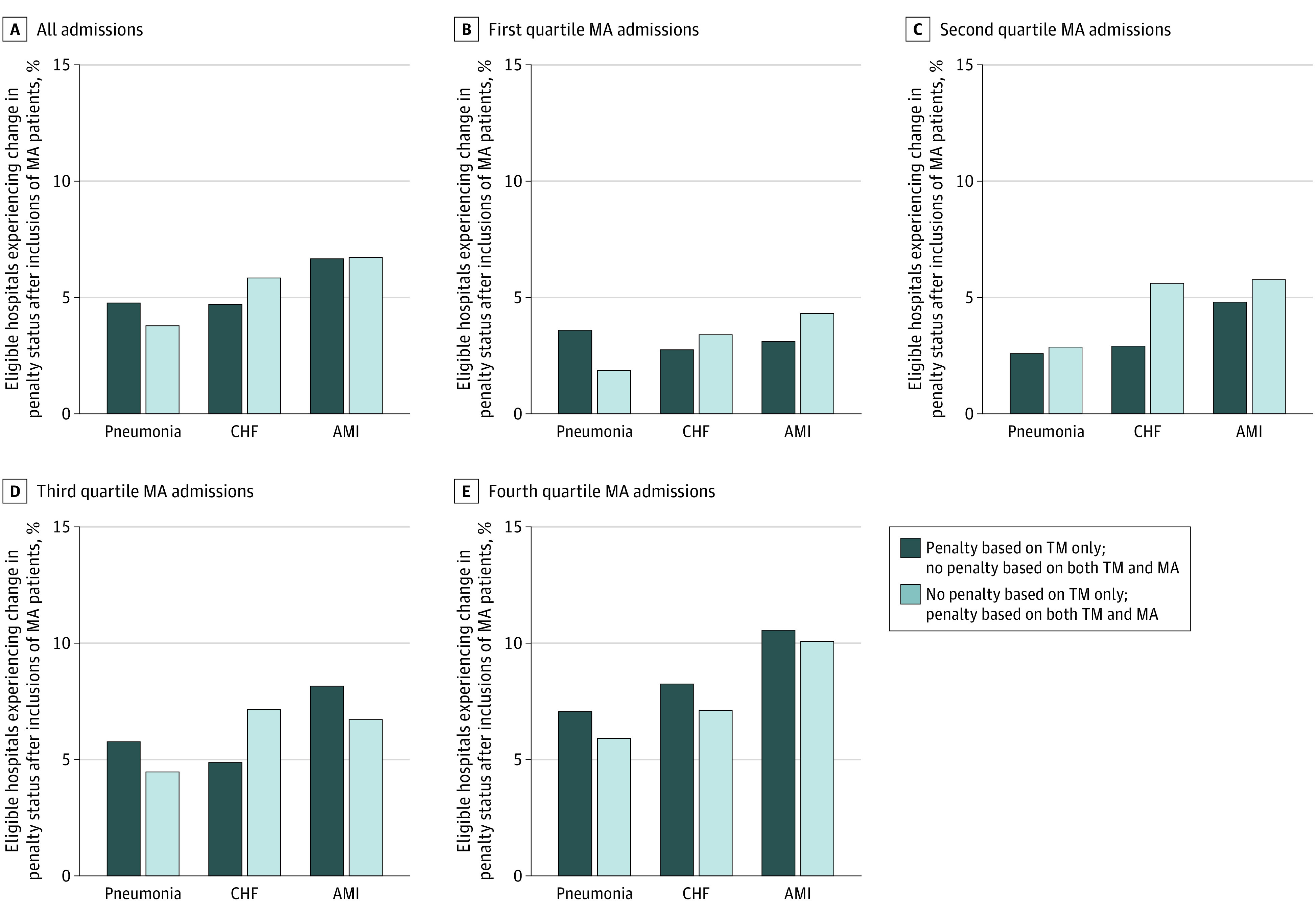
Changes in Hospital Penalty Status After Inclusion of Medicare Advantage (MA) Patients Eligible hospitals were limited to those with 25 or more admissions for a given index condition. AMI indicates acute myocardial infarction; CHF, congestive heart failure; TM, traditional Medicare.

The proportion of hospitals changing penalty status after inclusion of MA enrollees was 223 hospitals (13%) for AMI, 260 hospitals (11%) for CHF, and 237 hospitals (9%) for pneumonia (Figure 2). Of the 2820 hospitals admitting both TM and MA beneficiaries with 25 or more admissions for at least 1 of the conditions of AMI, CHF, and pneumonia, 635 (23%) had a change in their penalty status for at least 1 of the 3 conditions for both TM and MA data; of those, 326 (12%) changed from being penalized to not being penalized with the inclusion of MA, and 332 (12%) changed from not being penalized to being penalized. Across conditions, 229 hospitals (30%) in the upper quartile of MA admissions experienced a change in penalty status for at least 1 condition and, of those hospitals, 114 (50%) changed from being not penalized to being penalized (Figure 2).

## Discussion

In this analysis of nationwide data from TM and MA beneficiaries, we examined how inclusion of MA patients is associated with hospitals’ 30-day RSRRs and penalty status for AMI, CHF, and pneumonia, 3 conditions included in the CMS’s HRRP since its inception. When using data from each hospital’s both TM and MA patients to estimate the RSRRs, approximately 23% of hospitals changed their penalty status for at least 1 condition and 10% were no longer considered a low-performing outlier. These findings are a consequence of the correlation in readmission rates for MA and TM patients in the same hospital, larger differences for higher RSRRs when they are estimated using TM enrollees compared with all Medicare enrollees, and the high fraction of MA patients in some hospitals. Our results suggest that the inclusion of patients enrolled in MA plans in the HRRP would have material consequences for readmission penalties for US hospitals, especially those admitting large numbers of MA patients.

The CMS’s risk-adjustment model for 30-day readmission risk had comparable discrimination for both TM and MA patients. However, the number of hospitals that experience a change in readmission penalties would be substantially lower if the risk-adjustment model coefficients for TM patients were directly applied to MA patients. This finding suggests that the association between including MA patients and readmission penalties depends on how policy makers adapt existing risk-adjustment models for this new population. Thus, estimating the readmission risk model and calculating RSRRs for the entire Medicare population is associated with the performance of many hospitals; however, this association is substantially attenuated when a risk model that is based on TM enrollees is applied to both TM and MA enrollees. Moreover, as shown by our sensitivity analyses, it is possible that changes in hospital performance are not entirely explained by the addition of MA patients but it could reflect shrinkage to the national mean, especially for small hospitals with unstable readmission rates.

Previous studies have shown that hospital performance is not consistent across age and insurance groups. As the drivers of readmission are likely different between younger and older patients,^25^ hospital rankings based on older patients may not align with those derived from younger patients.^26^ To address this concern, we focused exclusively on older Medicare beneficiaries and extended prior work by assessing how readmission rates change when including Medicare beneficiaries who enrolled in the MA program. We further built on prior work^4^ by using a more precise measure of the type of insurance coverage^27^; specifically, we used Medicare administrative data to establish enrollment in an MA plan at the time of admission. We also examined data from all hospitals in the US and nearly all Medicare beneficiaries hospitalized with AMI, CHF, or pneumonia between 2011 and 2015.

Our study highlights the implications of including all Medicare patients in measuring hospital performance and implementing financial penalties. CMS’s current practice of relying exclusively on TM data was likely driven by the availability of a common data source and the construction of a valid and national risk-adjustment model rather than the increased importance of measuring quality for TM beneficiaries as compared with Medicare Advantage beneficiaries.^27^ As a result, the inclusion of TM data alone may incentivize hospitals to focus efforts to prevent readmission on these patients or to be unaware of readmission rates for other patients. Our findings demonstrate that the inclusion of MA data in deriving estimates of readmission is feasible and has material consequences for performance assessment.

As an alternative payment model, the MA program and its plans operate under strong financial incentives and have capabilities to manage beneficiaries’ use and transitions to postacute care, which could potentially affect subsequent readmissions. Similar incentives, however, exist for other alternative payment models that apply to TM patients (eg, accountable care organizations, bundled payments), yet the CMS does not exclude patients in these models from HRRP measures. The CMS’s goals in measuring hospital performance and reporting data on readmission rates to the broader public support using the entirety of each hospital’s Medicare population regardless of the type of Medicare coverage.

### Limitations

Our study has limitations. First, readmission rates for AMI, CHF, and pneumonia may not reflect the readmission rates of Medicare beneficiaries for other conditions at a given hospital.^27^ Although the Medicare Payment Advisory Commission has recommended the use of a hospital-wide readmission measure,^28^ this measure has not been adopted by CMS for the implementation of financial penalties, something that would require revision of the current penalty structure.^29^ Second, HEDIS records do not differentiate between planned and unplanned hospital admissions. Nevertheless, we have previously found that more than 90% of hospital readmissions are unplanned, and it is unlikely that our findings would change substantially had we excluded planned readmissions making up less than 10% of all readmissions.^9^ Third, we could not assess the validity of comorbid conditions reported for MA and TM patients in MedPAR. The number of comorbid conditions among hospitalized patients has increased over time in the TM population,^30^ whereas MA plans face incentives to inflate the number and type of diagnoses in CMS’s risk-adjusted payment model.^31^ However, we are unaware of evidence of differential coding of comorbid conditions in inpatient claims for TM and MA patients admitted to the same hospital. Fourth, we did not examine variation in readmission rates among hospital-owned MA plans, although the latter may have more efficient mechanisms to prevent readmissions. This is an important research question but is beyond the scope of the work presented here and should be examined in future research. Fifth, we were unable to estimate the changes in the penalty amounts for each hospital with the addition of MA patients because the base operating Diagnosis-Related Group payments are not publicly available for each hospital. Sixth, we have examined hospital performance for the 3 conditions used since the initiation of the HRRP. Given the nature of the MA program, it is reasonable to expect similar findings for other conditions in the current stratified HRRP; to confirm this, an updated analysis with more conditions could be conducted in the future. Seventh, to ensure that our approach replicates CMS’s methods, we included more than 1 index hospitalization for the same individual (if applicable) without clustering such hospitalizations at the person-level. As we have previously shown,^9^ accounting for such clustering (eg, through random-effects modeling) yields identical results with methods not accounting for clustering.

## Conclusions

This cohort study found that inclusion of data from MA patients changed the penalty status of a substantial fraction of US hospitals for at least 1 of 3 reported conditions. These findings highlight the need for CMS and other policy makers to consider incorporating outcomes of all hospitalized patients regardless of the source of insurance coverage in assessments of hospital quality.

## Notes

Supplement. **eAppendix.** Estimation of Risk-Standardized Readmission Rates **eTable 1.** Condition-Specific Comorbid Conditions Used in the CMS 30-Day Readmission Measures **eTable 2.** Characteristics of Traditional Medicare and Medicare Advantage Beneficiaries Hospitalized for Acute Myocardial Infarction, Heart Failure, or Pneumonia Between 2011 and 2015 **eTable 3.** Model Coefficients for 30-Day Readmission After Acute Myocardial Infarction in Traditional Medicare, Medicare Advantage, and All Medicare Patients **eTable 4.** Model Coefficients for 30-Day Readmission After Congestive Heart Failure in Traditional Medicare, Medicare Advantage, and All Medicare Patients **eTable 5.** Model Coefficients for 30-Day Readmission After Pneumonia in Traditional Medicare, Medicare Advantage, and All Medicare Patients **eTable 6.** Agreement in Hospital Rankings in 30-Day Readmission Rates After AMI, CHF, and Pneumonia for Traditional Medicare and All Enrollees (Traditional Medicare and Medicare Advantage); Predicted by TM Hierarchical Model **eTable 7.** Agreement in Hospital Rankings in 30-Day Readmission Rates After AMI, CHF, and Pneumonia for Traditional Medicare and All Enrollees (Traditional Medicare and Medicare Advantage) Where MA and TM Patients Were Proportionally Sampled According to Their Rate Such That the Overall Number of Individuals in Each Hospital Is the Same as the Size of the TM Population **eTable 8.** Changes in Outlier Status By Quartiles of % MA Admissions for AMI **eTable 9.** Changes in Outlier Status By Quartiles of % MA Admissions for CHF **eTable 10.** Changes in Outlier Status By Quartiles of % MA Admissions for Pneumonia **eFigure 1.** Flowchart of Eligible Hospitals for AMI, CHF, and Pneumonia **eFigure 2.** Receiver Operating Characteristic Curves for the 30-Day Readmission Model in TM, MA, and All Medicare **eFigure 3.** Distribution of Hospital-Specific 30-Day Risk Standardized Readmission Rates After AMI for Traditional Medicare, Medicare Advantage, and All Enrollees **eFigure 4.** Distribution of Hospital-Specific 30-Day Risk Standardized Readmission Rates after CHF for Traditional Medicare, Medicare Advantage, and all Enrollees **eFigure 5.** Distribution of Hospital-Specific 30-Day Risk Standardized Readmission Rates after Pneumonia for Traditional Medicare, Medicare Advantage, and All Enrollees **eFigure 6.** Correlation Between Hospital-Specific 30-Day Readmission Rates After AMI, CHF, and Pneumonia in Traditional Medicare and Both Traditional Medicare and Medicare Advantage Enrollees (Top); and Traditional Medicare and Medicare Advantage Enrollees (Bottom) **eFigure 7.** Bland-Altman Plots Comparing 30-Day RSRRs for Traditional Medicare Patients and All Medicare Patients **eFigure 8.** Hospital Performance by Condition Based on Traditional Medicare and All Medicare Patients
