# Supplementary material for: Association of Inclusion of Medicare Advantage Patients in Hospitals’ Risk-Standardized Readmission Rates, Performance, and Penalty Status
Source: JAMA Netw Open. 2021 Feb 17;4(2):e2037320. doi: 10.1001/jamanetworkopen.2020.37320 (PMC7890527; doi:10.1001/jamanetworkopen.2020.37320)
Supplement: Supplement. — eAppendix. Estimation of Risk-Standardized Readmission Rates eTable 1. Condition-Specific Comorbid Conditions Used in the CMS 30-Day Readmission Measures eTable 2. Characteristics of Traditional Medicare and Medicare Advantage Beneficiaries Hospitalized for Acute Myocardial Infarction, Heart Failure, or Pneumonia Between 2011 and 2015 eTable 3. Model Coefficients for 30-Day Readmission After Acute Myocardial Infarction in Traditional Medicare, Medicare Advantage, and All Medicare Patients eTable 4. Model Coefficients for 30-Day Readmission After Congestive Heart Failure in Traditional Medicare, Medicare Advantage, and All Medicare Patients eTable 5. Model Coefficients for 30-Day Readmission After Pneumonia in Traditional Medicare, Medicare Advantage, and All Medicare Patients eTable 6. Agreement in Hospital Rankings in 30-Day Readmission Rates After AMI, CHF, and Pneumonia for Traditional Medicare and All Enrollees (Traditional Medicare and Medicare Advantage); Predicted by TM Hierarchical Model eTable 7. Agreement in Hospital Rankings in 30-Day Readmission Rates After AMI, CHF, and Pneumonia for Traditional Medicare and All Enrollees (Traditional Medicare and Medicare Advantage) Where MA and TM Patients Were Proportionally Sampled According to Their Rate Such That the Overall Number of Individuals in Each Hospital Is the Same as the Size of the TM Population eTable 8. Changes in Outlier Status By Quartiles of % MA Admissions for AMI eTable 9. Changes in Outlier Status By Quartiles of % MA Admissions for CHF eTable 10. Changes in Outlier Status By Quartiles of % MA Admissions for Pneumonia eFigure 1. Flowchart of Eligible Hospitals for AMI, CHF, and Pneumonia eFigure 2. Receiver Operating Characteristic Curves for the 30-Day Readmission Model in TM, MA, and All Medicare eFigure 3. Distribution of Hospital-Specific 30-Day Risk Standardized Readmission Rates After AMI for Traditional Medicare, Medicare Advantage, and All Enrollees eFigure 4. Distribution of Hospital-S [file jamanetwopen-e2037320-s001.pdf]

Supplemental Online Content

Panagiotou OA, Voorhies KR, Keohane LM, et al. Association of inclusion of Medicare Advantage patients in hospitals' risk-standardized readmission rates, performance, and penalty status. *JAMA Netw Open*. 2021;4(2):e2037320. doi:10.1001/jamanetworkopen.2020.37320

eAppendix. Estimation of Risk-Standardized Readmission Rates

eTable 1. Condition-Specific Comorbid Conditions Used in the CMS 30-Day Readmission Measures

eTable 2. Characteristics of Traditional Medicare and Medicare Advantage Beneficiaries Hospitalized For Acute Myocardial Infarction, Heart Failure, or Pneumonia Between 2011 and 2015

eTable 3. Model Coefficients for 30-Day Readmission After Acute Myocardial Infarction in Traditional Medicare, Medicare Advantage, and All Medicare Patients

eTable 4. Model Coefficients for 30-Day Readmission After Congestive Heart Failure in Traditional Medicare, Medicare Advantage, and All Medicare Patients

eTable 5. Model Coefficients for 30-Day Readmission After Pneumonia in Traditional Medicare, Medicare Advantage, and All Medicare Patients

eTable 6. Agreement in Hospital Rankings in 30-Day Readmission Rates After AMI, CHF, and Pneumonia for Traditional Medicare and All Enrollees (Traditional Medicare and Medicare Advantage); Predicted by TM Hierarchical Model

eTable 7. Agreement in Hospital Rankings in 30-Day Readmission Rates After AMI, CHF, and Pneumonia for Traditional Medicare and All Enrollees (Traditional Medicare and Medicare Advantage) Where MA and TM Patients Were Proportionally Sampled According to Their Rate Such That the Overall Number of Individuals in Each Hospital is the Same as the Size of the TM Population

eTable 8. Changes in Outlier Status By Quartiles of % MA Admissions for AMI

eTable 9. Changes in Outlier Status By Quartiles of % MA Admissions for CHF

eTable 10. Changes in Outlier Status By Quartiles of % MA Admissions for Pneumonia

eFigure 1. Flowchart of Eligible Hospitals for AMI, CHF, and Pneumonia

eFigure 2. Receiver Operating Characteristic Curves for the 30-Day Readmission Model in TM, MA, and All Medicare

eFigure 3. Distribution of Hospital-Specific 30-Day Risk Standardized Readmission Rates After AMI for Traditional Medicare, Medicare Advantage, and All Enrollees

© 2021 Panagiotou OA et al. *JAMA Network Open.*

eFigure 4. Distribution of Hospital-Specific 30-Day Risk Standardized Readmission Rates after CHF for Traditional Medicare, Medicare Advantage, and all Enrollees

eFigure 5. Distribution of Hospital-Specific 30-Day Risk Standardized Readmission Rates after Pneumonia for Traditional Medicare, Medicare Advantage, and All Enrollees

eFigure 6. Correlation Between Hospital-Specific 30-Day Readmission Rates After AMI, CHF, and Pneumonia in Traditional Medicare and Both Traditional Medicare and Medicare Advantage Enrollees (Top); and Traditional Medicare and Medicare Advantage Enrollees (Bottom)

eFigure 7. Bland-Altman Plots Comparing 30-Day RSRRs for Traditional Medicare Patients and All Medicare Patients

eFigure 8. Hospital Performance by Condition Based on Traditional Medicare and All Medicare Patients

This supplemental material has been provided by the authors to give readers additional information about their work.

© 2021 Panagiotou OA et al. *JAMA Network Open.*

eAppendix. Estimation of Risk-Standardized Readmission Rates

The estimation of the hospital-specific 30-day risk-standardized readmission rates follows the technical specifications used by the Centers for Medicare and Medicaid Services (CMS) and are available at: https://www.cms.gov/Medicare/Quality-Initiatives-Patient-Assessment-Instruments/HospitalQualityInits/Measure-Methodology.

We used a hierarchical generalized linear model to account for within-hospital correlation of the observed outcome. This modeling approach satisfies a key assumption of the HRRP, i.e. underlying differences in quality across hospitals lead to systematic differences in outcomes. We estimated separate models for patients discharged after AMI, CHF, and pneumonia. The goal is to model the probability of readmission given a patient's age, sex and comorbid conditions (specific for each of AMI, CHF, pneumonia; see eTable 2) with an intercept for the hospital-specific random effect. Models were estimated separately for TM beneficiaries, MA beneficiaries, and the combined sample of TM and MA beneficiaries.

Each hospital's condition-specific 30-day RSRR for each risk model coefficients' estimates was estimated as the ratio of its "predicted" and "expected" readmissions standardized to the national observed readmission rate for that condition and population (TM, MA, both); 95% confidence intervals (CIs) were computed using the bootstrap procedure. We estimated the "predicted" readmission risk using a multilevel logistic regression model with specific intercept for each hospital effect and linear additive adjustments for the covariates described above. We estimated the "expected" readmission risk similarly but relied on the average of the hospital-specific intercepts.

© 2021 Panagiotou OA et al. *JAMA Network Open.*

Given the binary nature of the outcome (readmitted within 30 days: yes/no), we used a hierarchical generalized linear model with a logit link function as follows:

$$h(Y_{ij}) = \alpha_i + \beta \boldsymbol{Z}_{ij}$$

$$\alpha_i = \mu + \omega_i \text{ with } \omega_i \sim N(0, \tau^2)$$

$h(.)$ is a logit link, Y_{ij} is whether the jth patient in the ith hospital was readmitted (1: readmitted, 0: otherwise); α_i represents the hospital-specific intercept, $\mathbf{Z}_{ij}$ = (Z_{1ij},Z_{2ij}, ... , Z_{pij}) the patient-specific covariates, μ is the average hospital intercept across all hospitals in the sample, and τ^2 is the between-hospital variance component. This model separates within-hospital variation from between-hospital variation.

To calculate the standardized measure (RSRR) for each hospital, we first computed the ratio of the predicted number of readmissions to the expected number of readmissions and multiplied this ratio by the national observed readmission rate $\bar{y}$:

Predicted readmissions: $\widehat{y_{ij}}(\boldsymbol{Z}_{ij}) = h^{-1}(\hat{\alpha}_i + \hat{\beta}\boldsymbol{Z}_{ij})$

Expected readmissions: $\widehat{e_{ij}}(\boldsymbol{Z}_{ij}) = h^{-1}(\mu + \hat{\beta}\boldsymbol{Z}_{ij})$

Standardized measure: $RSRR_i = \frac{\sum_{j-1}^{n_i} \widehat{y_{ij}}(\boldsymbol{Z}_{ij})}{\sum_{j-1}^{n_i} \widehat{e_{ij}}(\boldsymbol{Z}_{ij})} \times \bar{y}$

where n_i is the number of index hospitalizations for the ith hospital.

Confidence intervals around the RSRR were computed using bootstrap.

© 2021 Panagiotou OA et al. *JAMA Network Open.*

We used the $\bar{y}$ that was applicable to each condition (AMI, CHF, pneumonia) and each population, e.g. a different national observed readmission rate for TM beneficiaries with AMI and a different national observed readmission rate for MA beneficiaries with AMI etc.

Overall, for each hospital we computed the following RSRRS as applicable:

1. RSRR for TM beneficiaries with AMI
2. RSRR for MA beneficiaries with AMI
3. RSRR for all Medicare (i.e. both TM and MA) beneficiaries with AMI
4. RSRR for TM beneficiaries with CHF
5. RSRR for MA beneficiaries with CHF
6. RSRR for all Medicare (i.e. both TM and MA) beneficiaries with CHF
7. RSRR for TM beneficiaries with pneumonia
8. RSRR for MA beneficiaries with pneumonia
9. RSRR for all Medicare (i.e. both TM and MA) beneficiaries with pneumonia

© 2021 Panagiotou OA et al. *JAMA Network Open.*

eTable 1. Condition-specific comorbid conditions used in the CMS 30-day readmission measures

Shown are the number of individuals with comorbidities and the prevalence rates.

	**Acute Myocardial Infarction (N = 1,040,975)**		**Congestive Heart Failure (N = 1,766,929)**		**Pneumonia (N = 2,611,909)**	
**Comorbid conditions, N (%)**	**Traditional Medicare**	**Medicare Advantage**	**Traditional Medicare**	**Medicare Advantage**	**Traditional Medicare**	**Medicare Advantage**
Acute coronary syndrome	77,854 (10.4)	26,787 (9.1)	107,078 (8.1)	35,644 (7.8)	68,220 (3.4)	19,295 (3.2)
Iron deficiency or other unspecified anemias and blood disease	237,034 (31.7)	84,986 (28.7)	553,515 (41.7)	175,393 (38.4)	776,888 (38.5)	213,713 (35.0)
Coronary atherosclerosis, angina, or other chronic ischemic heart disease	526,471 (70.4)	201,684 (68.2)	723,577 (54.5)	237,470 (51.9)	671,210 (33.3)	185,363 (30.3)
Angina pectoris / old myocardial infarction	110,175 (14.7)	43,534 (14.7)				
Anterior myocardial infarction	55,599 (7.4)	22,576 (7.6)				
Asthma	21,994 (2.9)	8,866 (3.0)	50,496 (3.8)	18,025 (3.9)	85,561 (4.2)	27,315 (4.5)
Cardiopulmonary-respiratory failure and shock			157,180 (11.8)	50,192 (11.0)		
Cerebrovascular disease	37,585 (5.0)	12,201 (4.1)				
Congestive heart failure	103,998 (13.9)	33,080 (11.2)	410,143 (30.9)	127,647 (27.9)	322,609 (16.0)	85,006 (13.9)
COPD	155,116 (20.7)	54,667 (18.5)	434,129 (32.7)	139,134 (30.4)	711,851 (35.3)	204,005 (33.4)
Decubitus skin ulcer	18,706 (2.5)	5,810 (2.0)	69,417 (5.2)	20,679 (4.5)	93,275 (4.6)	22,937 (3.8)
Dementia/Other specified brain disorders	94,250 (12.6)	27,551 (9.3)	207,486 (15.6)	53,970 (11.8)	501,523 (24.9)	114,127 (18.7)
Depression			173,541 (13.1)	49,792 (10.9)		
Diabetes mellitus or diabetic complications	255,110 (34.1)	102,646 (34.7)	523,571 (39.4)	186,039 (40.7)	595,479 (29.5)	180,222 (29.5)
On dialysis	11,327 (1.5)	2,852 (1.0)	25,095 (1.9)	5,592 (1.2)	30,461 (1.5)	6,624 (1.1)
Drug/alcohol abuse			95,838 (7.2)	35,050 (7.7)	165,216 (8.2)	53,938 (8.8)
Fluid and electrolyte disorders	93,428 (12.5)	28,266 (9.6)	303,439 (22.9)	89,112 (19.5)	398,300 (19.7)	99,764 (16.3)
Coronary artery bypass grafting	120,889 (16.2)	49,906 (16.9)	148,522 (11.2)	51,320 (11.2)	105,132 (5.2)	31,258 (5.1)
History of infection	36,714 (4.9)	10,779 (3.6)			197,596 (9.8)	48,205 (7.9)
Percutaneous transluminal coronary angioplasty	340,250 (45.5)	139,693 (47.2)				

© 2021 Panagiotou OA et al. *JAMA Network Open.*

Liver or biliary disease			24,026 (1.8)	8,284 (1.8)		
Fibrosis of lung or other chronic lung disorders			55,627 (4.2)	16,807 (3.7)	111,824 (5.5)	32,364 (5.3)
Other major cancers					136,033 (6.7)	39,569 (6.5)
Major psychiatric disorders			42,775 (3.2)	14,150 (3.1)	99,997 (5.0)	26,510 (4.3)
Protein-calorie malnutrition	33,589 (4.5)	11,346 (3.8)	89,861 (6.8)	28,004 (6.1)	238,158 (11.8)	67,186 (11.0)
Metastatic cancer/Acute leukemia	8,980 (1.2)	2,984 (1.0)	18,466 (1.4)	5,690 (1.2)	65,444 (3.2)	20,081 (3.3)
Nephritis			22,823 (1.7)	8,170 (1.8)		
Other cancers	43,142 (5.8)	14,721 (5.0)	100,577 (7.6)	31,909 (7.0)		
Other gastrointestinal disorders			498,674 (37.6)	154,974 (33.9)	818,686 (40.6)	224,932 (36.8)
Other unspecified heart disease			86,528 (6.5)	26,281 (5.7)		
Other injuries					131,432 (6.5)	32,355 (5.3)
Other lung disorders					222,283 (11.0)	59,253 (9.7)
Inferior, lateral, or posterior MI	87,719 (11.7)	36,414 (12.3)				
Other psychiatric disorders			129,014 (9.7)	39,342 (8.6)	218,863 (10.9)	58,831 (9.6)
Other urinary tract disorders	58,977 (7.9)	19,772 (6.7)	140,724 (10.6)	42,545 (9.3)	173,434 (8.6)	45,156 (7.4)
Peptic ulcer, hemorrhage and other specified gastrointestinal disorders			83,875 (6.3)	24,943 (5.5)		
Hemiplegia, paraplegia, paralysis or functional disability	20,783 (2.8)	6,409 (2.2)	49,787 (3.8)	14,589 (3.2)	99,041 (4.9)	24,280 (4.0)
Pleural effusion/pneumothorax					58,063 (2.9)	15,673 (2.6)
Pneumonia	91,579 (12.2)	28,889 (9.8)	310,166 (23.4)	90,398 (19.8)	1,329,833 (65.9)	386,613 (63.3)
Renal failure	101,898 (13.6)	34,293 (11.6)	336,410 (25.3)	106,767 (23.3)	332,518 (16.5)	91,455 (15.0)
Cardiopulmonary-respiratory failure and shock; respiratory arrest					198,616 (9.8)	56,681 (9.3)
Specified arrhythmias and other heart rhythm disorders	99,559 (13.3)	31,423 (10.6)	379,906 (28.6)	115,737 (25.3)	355,641 (17.6)	93,981 (15.4)
Septicemia/shock					102,032 (5.1)	26,777 (4.4)
Lung / Upper gastrointestinal / Other severe cancers					73,097 (3.6)	22,986 (3.8)
Severe hematological disorders			20,079 (1.5)	5,257 (1.1)	30,253 (1.5)	7,546 (1.2)
Stroke	11,273 (1.5)	3,746 (1.3)	26,746 (2.0)	8,358 (1.8)	49,920 (2.5)	13,401 (2.2)

© 2021 Panagiotou OA et al. *JAMA Network Open.*

Respirator/Tracheostomy dependence					9,845 (0.5)	2,720 (0.4)
Urinary tract infection					229,055 (11.4)	52,949 (8.7)
Valvular or rheumatic heart disease	138,709 (18.5)	47,633 (16.1)	408,417 (30.8)	130,156 (28.5)	219,356 (10.9)	60,312 (9.9)
Vascular or circulatory disease	78,012 (10.4)	26,643 (9.0)	230,179 (17.3)	73,095 (16.0)	262,282 (13.0)	73,533 (12.0)
Vertebral fractures					38,980 (1.9)	10,236 (1.7)

Data are shown at the beneficiary level.

An empty cell means that a comorbid condition (row) is not included the risk-adjustment model used by the Centers for Medicare and Medicaid Services to estimate 30-day readmission rate for a given condition (column).

© 2021 Panagiotou OA et al. *JAMA Network Open.*

eTable 2. Characteristics of Traditional Medicare and Medicare Advantage beneficiaries hospitalized for acute myocardial infarction, heart failure, or pneumonia between 2011 and 2015*

	AMI (N=1,131,506)		CHF (N=2,532,835)		Pneumonia(N=3,216,363)	
	**TM**	**MA**	**TM**	**MA**	**TM**	**MA**
Index admissions with alive discharges, N						
All	812,656	318,850	1,899,272	633,563	2,499,778	716,585
2011	169,187	55,383	405,930	110,834	510,450	117,418
2012	168,696	59,697	385,385	114,848	492,380	123,719
2013	164,380	64,568	377,039	125,338	499,272	140,298
2014	161,145	69,656	378,046	140,008	476,994	151,415
2015	149,248	69,546	352,872	142,535	520,682	183,735
Hospitals, N	3,167	3,167	3,838	3,838	4,010	4,010
Beneficiaries	748,033	295,928	1,327,551	457,341	2,017,020	610,790
Age, mean (SD), y	78.6 (8.3)	77.5 (7.9)	81 (8.3)	79.8 (8.1)	80.7 (8.5)	79.6 (8.2)
Male Gender, (%)	387,431 (51.8)	158,506 (53.6)	591,696 (44.6)	213,838 (46.8)	919,869 (45.6)	289,440 (47.4)
Race, N (%)						
White	631,805 (84.5)	229,107 (77.4)	1,088,546 (82.0)	338,427 (74.0)	1,699,731 (84.3)	480,013 (78.6)
Black	57,322 (7.7)	28,969 (9.8)	141,597 (10.7)	67,035 (14.7)	157,156 (7.8)	58,592 (9.6)
Hispanic	36,039 (4.8)	27,448 (9.3)	63,015 (4.7)	38,803 (8.5)	98,087 (4.9)	50,110 (8.2)
Other	20,802 (2.8)	9,603 (3.2)	32,162 (2.4)	12,353 (2.7)	58,405 (2.9)	21,093 (3.5)

* No formal (i.e. null-hypothesis testing-based) comparison between the two groups was performed for baseline characteristics. AMI denotes acute myocardial infarction, CHF congestive heart failure, MA Medicare Advantage, SD standard deviation, and TM Traditional Medicare.

† Calculated at the beneficiary level.

© 2021 Panagiotou OA et al. *JAMA Network Open.*

eTable 3. Model coefficients for 30-day readmission after acute myocardial infarction in traditional Medicare, Medicare Advantage, and all Medicare patients

	Traditional Medicare			Medicare Advantage			All Medicare		
**Variable**	**OR**	**LCI**	**UCI**	**OR**	**LCI**	**UCI**	**OR**	**LCI**	**UCI**
Acute coronary syndrome	0.99	1.00	0.97	0.97	1.00	0.94	0.98	1.00	0.97
Age (greater than 65)	1.01	1.01	1.01	1.01	1.01	1.01	1.01	1.01	1.01
Iron deficiency or other unspecified anemias and blood disease	1.21	1.23	1.20	1.28	1.31	1.25	1.23	1.25	1.22
Angina pectoris/old MI	1.04	1.06	1.02	1.01	1.04	0.98	1.03	1.05	1.02
Anterior MI	1.14	1.17	1.12	1.20	1.25	1.16	1.16	1.18	1.14
Asthma	0.99	1.03	0.96	0.99	1.05	0.94	0.99	1.02	0.97
Cerebrovascular disease	1.04	1.07	1.01	1.07	1.12	1.03	1.05	1.07	1.03
Congestive heart failure	1.22	1.24	1.20	1.18	1.22	1.14	1.21	1.23	1.19
Chronic Ischemic Heart Disease	0.96	0.97	0.94	0.92	0.95	0.90	0.95	0.96	0.94
COPD	1.25	1.27	1.23	1.22	1.25	1.19	1.24	1.26	1.22
Decubitus skin ulcer	1.14	1.18	1.11	1.05	1.12	0.99	1.12	1.15	1.09
Dementia/Other specified brain disorders	0.92	0.94	0.90	0.93	0.96	0.90	0.92	0.94	0.91
Diabetes mellitus or diabetic complications	1.19	1.20	1.17	1.22	1.25	1.19	1.20	1.21	1.18
On dialysis	1.48	1.54	1.43	1.52	1.63	1.42	1.48	1.53	1.43
Fluid and electrolyte disorders	1.08	1.10	1.06	1.11	1.15	1.07	1.08	1.10	1.07
CABG	0.92	0.94	0.91	1.03	1.06	1.00	0.95	0.96	0.94
History of infection	1.07	1.09	1.04	1.05	1.10	1.01	1.06	1.09	1.04
PTCA	0.87	0.88	0.85	0.81	0.83	0.79	0.85	0.86	0.84
Gender = Male	0.94	0.95	0.93	0.96	0.98	0.94	0.95	0.96	0.94
Protein-calorie malnutrition	0.98	1.01	0.96	1.12	1.17	1.07	1.02	1.04	0.99
Metastatic cancer/Acute leukemia	1.13	1.19	1.08	1.13	1.23	1.03	1.13	1.18	1.08
Other cancers	1.13	1.16	1.11	1.13	1.18	1.09	1.13	1.16	1.11
Inferior, lateral, or posterior MI	0.93	0.94	0.91	0.91	0.94	0.88	0.92	0.94	0.90
Other urinary tract disorders	1.08	1.10	1.06	1.09	1.13	1.05	1.08	1.10	1.06
Hemiplegia, paraplegia, paralysis or functional disability	1.08	1.12	1.05	1.06	1.12	1.00	1.08	1.11	1.05
Pneumonia	1.09	1.11	1.07	1.14	1.18	1.11	1.10	1.12	1.09
Renal failure	1.14	1.17	1.12	1.16	1.20	1.12	1.15	1.17	1.13
Specified arrhythmias and other heart rhythm disorders	1.05	1.07	1.03	1.07	1.10	1.04	1.05	1.07	1.04
Stroke	0.92	0.96	0.87	1.03	1.11	0.95	0.94	0.98	0.91

© 2021 Panagiotou OA et al. *JAMA Network Open.*

Valvular or rheumatic heart disease	1.15	1.17	1.14	1.13	1.15	1.10	1.15	1.16	1.13
Vascular or circulatory disease	1.10	1.12	1.08	1.10	1.14	1.07	1.10	1.12	1.08

© 2021 Panagiotou OA et al. *JAMA Network Open.*

eTable 4. Model coefficients for 30-day readmission after congestive heart failure in traditional Medicare, Medicare Advantage, and all Medicare patients

	Traditional Medicare			Medicare Advantage			All Medicare		
**Variable**	**OR**	**LCI**	**UCI**	**OR**	**LCI**	**UCI**	**OR**	**LCI**	**UCI**
Acute coronary syndrome	1.07	1.06	1.09	1.06	1.04	1.08	1.07	1.06	1.08
Age (greater than 65)	1.00	0.99	1.00	0.99	0.99	0.99	0.99	0.99	1.00
Iron deficiency or other unspecified anemias and blood disease	1.10	1.09	1.11	1.11	1.09	1.12	1.10	1.09	1.11
Angina/Old MI/Chronic Ischemic Heart Disease	1.01	1.00	1.02	1.00	0.99	1.01	1.00	1.00	1.01
Asthma	0.98	0.96	0.99	0.97	0.95	1.00	0.98	0.96	0.99
Cardiopulmonary-respiratory failure and shock	1.07	1.06	1.08	1.05	1.03	1.07	1.07	1.06	1.08
Congestive heart failure	1.12	1.11	1.13	1.12	1.10	1.15	1.12	1.11	1.13
COPD	1.13	1.12	1.14	1.13	1.11	1.14	1.13	1.12	1.14
Decubitus skin ulcer	1.12	1.10	1.13	1.12	1.09	1.15	1.12	1.11	1.13
Dementia/Other specified brain disorders	0.96	0.95	0.97	0.94	0.92	0.96	0.95	0.94	0.96
Depression	1.00	0.99	1.01	1.01	0.99	1.03	1.01	1.00	1.02
Diabetes mellitus or diabetic complications	1.05	1.04	1.05	1.04	1.03	1.05	1.04	1.04	1.05
On dialysis	1.26	1.23	1.28	1.25	1.20	1.30	1.25	1.23	1.27
Drug/alcohol abuse	1.04	1.03	1.06	1.02	1.00	1.04	1.04	1.03	1.05
Fluid and electrolyte disorders	1.08	1.07	1.09	1.10	1.08	1.12	1.08	1.08	1.09
CABG	1.03	1.02	1.04	1.01	1.00	1.03	1.03	1.02	1.03
Liver or biliary disease	1.10	1.08	1.12	1.12	1.08	1.16	1.10	1.08	1.13
Fibrosis of lung or other chronic lung disorders	1.05	1.04	1.07	1.09	1.06	1.12	1.06	1.05	1.08
Major psychiatric disorders	1.04	1.03	1.06	1.09	1.05	1.12	1.05	1.04	1.07
Gender = Male	1.00	1.00	1.01	1.01	1.00	1.03	1.01	1.00	1.01
Protein-calorie malnutrition	1.04	1.02	1.05	1.07	1.04	1.09	1.05	1.03	1.06
Metastatic cancer/Acute leukemia	1.10	1.07	1.14	1.10	1.04	1.16	1.10	1.07	1.13
Nephritis	1.10	1.08	1.12	1.12	1.08	1.16	1.11	1.09	1.12
Other cancers	1.08	1.06	1.09	1.06	1.04	1.09	1.07	1.06	1.09
Other gastrointestinal disorders	1.02	1.02	1.03	1.03	1.01	1.04	1.03	1.02	1.03
Other unspecified heart disease	1.00	0.99	1.01	0.99	0.97	1.02	1.00	0.99	1.01
Other psychiatric disorders	1.05	1.04	1.06	1.06	1.04	1.08	1.05	1.04	1.06
Other urinary tract disorders	1.06	1.04	1.07	1.07	1.05	1.09	1.06	1.05	1.07

© 2021 Panagiotou OA et al. *JAMA Network Open.*

Peptic ulcer, hemorrhage and other specified GI disorders	1.07	1.06	1.09	1.04	1.02	1.07	1.07	1.05	1.08
Hemiplegia, paraplegia, paralysis or functional disability	1.03	1.01	1.04	1.05	1.02	1.09	1.03	1.02	1.05
Pneumonia	1.02	1.01	1.03	1.03	1.01	1.04	1.02	1.01	1.03
Renal failure	1.14	1.13	1.15	1.14	1.12	1.16	1.14	1.13	1.15
Specified arrhythmias and other heart rhythm disorders	1.01	1.00	1.02	1.02	1.00	1.04	1.01	1.00	1.02
Severe hematological disorders	1.22	1.19	1.25	1.20	1.14	1.26	1.22	1.19	1.24
Stroke	0.98	0.96	1.01	0.96	0.91	1.00	0.98	0.96	1.00
Valvular or rheumatic heart disease	1.05	1.04	1.06	1.04	1.03	1.05	1.05	1.04	1.06
Vascular or circulatory disease	1.06	1.05	1.07	1.06	1.05	1.08	1.06	1.06	1.07

© 2021 Panagiotou OA et al. *JAMA Network Open.*

eTable 5. Model coefficients for 30-day readmission after pneumonia in traditional Medicare, Medicare Advantage, and all Medicare patients

	**Traditional Medicare**			**Medicare Advantage**			**All Medicare**		
**Variable**	**OR**	**LCI**	**UCI**	**OR**	**LCI**	**UCI**	**OR**	**LCI**	**UCI**
Acute coronary syndrome	1.05	1.03	1.06	1.02	0.99	1.06	1.04	1.03	1.06
Age (greater than 65)	1.00	0.99	1.00	1.00	0.99	1.00	1.00	1.00	1.00
Iron deficiency or other unspecified anemias and blood disease	1.16	1.15	1.17	1.18	1.16	1.19	1.16	1.15	1.17
Angina/Old MI/Chronic Ischemic Heart Disease	1.05	1.04	1.06	1.07	1.05	1.09	1.05	1.05	1.06
Asthma	0.93	0.92	0.95	0.93	0.90	0.96	0.93	0.92	0.95
Congestive heart failure	1.16	1.15	1.18	1.17	1.15	1.20	1.17	1.15	1.18
COPD	1.16	1.15	1.17	1.13	1.11	1.15	1.15	1.15	1.16
Decubitus skin ulcer	1.13	1.11	1.14	1.16	1.13	1.20	1.13	1.12	1.15
Dementia/Other specified brain disorders	0.95	0.94	0.95	0.93	0.92	0.95	0.94	0.93	0.95
Diabetes mellitus or diabetic complications	1.08	1.08	1.09	1.07	1.06	1.09	1.08	1.07	1.09
On dialysis	1.34	1.32	1.37	1.29	1.23	1.35	1.33	1.30	1.36
Drug/alcohol abuse	1.00	0.99	1.01	1.00	0.98	1.02	1.00	0.99	1.01
Fluid and electrolyte disorders	1.10	1.09	1.11	1.10	1.08	1.13	1.10	1.09	1.11
CABG	0.99	0.98	1.00	0.98	0.95	1.00	0.99	0.97	1.00
History of infection	1.09	1.08	1.11	1.08	1.05	1.10	1.09	1.08	1.10
Fibrosis of lung or other chronic lung disorders	1.09	1.08	1.11	1.11	1.08	1.14	1.09	1.08	1.11
Other major cancers	1.10	1.08	1.11	1.12	1.09	1.15	1.10	1.09	1.11
Major psychiatric disorders	1.05	1.03	1.06	1.05	1.02	1.08	1.05	1.03	1.06
Gender = Male	1.06	1.06	1.07	1.06	1.05	1.08	1.06	1.06	1.07
Protein-calorie malnutrition	1.06	1.05	1.07	1.10	1.08	1.13	1.07	1.06	1.08
Metastatic cancer/Acute leukemia	1.15	1.12	1.17	1.09	1.05	1.14	1.13	1.11	1.15
Other gastrointestinal disorders	1.05	1.04	1.05	1.04	1.02	1.05	1.04	1.04	1.05
Other injuries	1.01	1.00	1.02	1.02	0.99	1.04	1.01	1.00	1.02
Other lung disorders	1.02	1.00	1.03	1.02	0.99	1.04	1.01	1.00	1.02
Other psychiatric disorders	1.04	1.03	1.05	1.07	1.05	1.09	1.05	1.04	1.06
Other urinary tract disorders	1.04	1.03	1.05	1.04	1.01	1.06	1.04	1.03	1.05
Hemiplegia, paraplegia, paralysis or functional disability	1.07	1.06	1.09	1.08	1.05	1.12	1.07	1.06	1.09
Pleural effusion/pneumothorax	1.05	1.03	1.06	1.04	1.00	1.07	1.04	1.03	1.06
Pneumonia	0.85	0.85	0.86	0.82	0.81	0.83	0.84	0.84	0.85
Renal failure	1.11	1.10	1.13	1.11	1.09	1.13	1.11	1.10	1.12
Cardiopulmonary-respiratory failure and shock; respiratory arrest	1.16	1.15	1.17	1.18	1.16	1.21	1.16	1.15	1.18

© 2021 Panagiotou OA et al. *JAMA Network Open.*

Specified arrhythmias and other heart rhythm disorders	1.05	1.04	1.06	1.06	1.04	1.08	1.05	1.05	1.06
Septicemia/shock	1.06	1.05	1.07	1.05	1.02	1.07	1.06	1.05	1.07
Lung/Upper GI/Other severe cancers	1.24	1.22	1.27	1.28	1.24	1.32	1.25	1.23	1.27
Severe hematological disorders	1.30	1.27	1.33	1.32	1.26	1.39	1.31	1.28	1.33
Stroke	0.99	0.97	1.01	1.04	1.00	1.09	1.00	0.98	1.02
Respirator/Tracheostomy dependence	1.14	1.11	1.18	1.30	1.22	1.38	1.17	1.14	1.20
Urinary tract infection	1.05	1.04	1.06	1.06	1.03	1.08	1.05	1.04	1.06
Valvular or rheumatic heart disease	1.08	1.06	1.09	1.09	1.06	1.11	1.08	1.07	1.09
Vascular or circulatory disease	1.06	1.05	1.07	1.08	1.06	1.10	1.06	1.05	1.07
Vertebral fractures	1.07	1.05	1.10	1.10	1.06	1.15	1.08	1.06	1.10

© 2021 Panagiotou OA et al. *JAMA Network Open.*

eTable 6. Agreement in Hospital Rankings in 30-Day Readmission Rates after AMI, CHF, and pneumonia for Traditional Medicare and All Enrollees (Traditional Medicare and Medicare Advantage); Predicted by TM hierarchical model

**Hospital 30-Day RSRR in TM**	**Hospitals, N**	**Hospital 30-Day RSRR in MA & TM**		
		Worse than expected	As expected	Better than expected
**AMI**	*N*	*67*	*3,054*	*46*
Worse than expected	*68*	64	**4**	**0**
As expected	*3,051*	**3**	3,047	**1**
Better than expected	*48*	**0**	**3**	45
**CHF**	*N*	*241*	*3,424*	*173*
Worse than expected	*242*	233	**9**	**0**
As expected	*3,417*	**8**	3,405	**4**
Better than expected	*179*	**0**	**10**	169
**Pneumonia**	*N*	*368*	*3,435*	*207*
Worse than expected	*370*	361	**9**	**0**
As expected	*3,421*	**7**	3,410	**4**
Better than expected	*219*	**0**	**16**	203

Of 486 hospitals with worse than expected readmission rates for at least one condition according to TM data alone, 22 (5%) were no longer worse than expected after the inclusion of MA data with model estimates based on TM patients.

© 2021 Panagiotou OA et al. *JAMA Network Open.*

eTable 7. Agreement in Hospital Rankings in 30-Day Readmission Rates after AMI, CHF, And Pneumonia for Traditional Medicare and All Enrollees (Traditional Medicare and Medicare Advantage) where MA and TM Patients Were Proportionally Sampled According to their Rate Such That the Overall Number of Individuals in Each Hospital is the Same as the Size of the TM Population.

**Hospital 30-Day RSRR in TM**	**Hospitals, N**	**Hospital 30-Day RSRR in MA & TM**		
**AMI**				
		Worse than expected	As expected	Better than expected
	N	*69*	*3,048*	*50*
Worse than expected	*51*	44	**7**	**0**
As expected	*3,078*	**25**	3,038	**15**
Better than expected	*38*	**0**	**3**	35
**CHF**				
		Worse than expected	As expected	Better than expected
	N	*260*	*3,400*	*178*
Worse than expected	*198*	180	**18**	**0**
As expected	*3,497*	**80**	3,364	**53**
Better than expected	*143*	**0**	**18**	125
**Pneumonia**				
		Worse than expected	As expected	Better than expected
	N	*403*	*3,380*	*227*
Worse than expected	*353*	322	**31**	**0**
As expected	*3,462*	**81**	3,323	**58**
Better than expected	*195*	**0**	**26**	169

© 2021 Panagiotou OA et al. *JAMA Network Open.*

eTable 8. Changes in outlier status by quartiles of % MA admissions for AMI

**% MA Admissions**	**Hospital 30-Day RSRR in TM**	**Hospitals, N**	**Hospital 30-Day RSRR in MA & TM**		
<**11.4%**			Worse than expected	As expected	Better than expected
		N	*10*	*771*	*11*
	Worse than expected	*8*	8	**0**	**0**
	As expected	*775*	**2**	771	**2**
	Better than expected	*9*	**0**	**0**	9
**11.4%-20.9%**			Worse than expected	As expected	Better than expected
		N	*21*	*750*	*20*
	Worse than expected	*17*	15	**2**	**0**
	As expected	*764*	**6**	748	**10**
	Better than expected	*10*	**0**	**0**	10
**20.9%-33.7%**			Worse than expected	As expected	Better than expected
		N	*38*	*725*	*29*
	Worse than expected	*23*	19	**4**	**0**
	As expected	*752*	**19**	720	**13**
	Better than expected	*17*	**0**	**1**	16
≥33.7%			Worse than expected	As expected	Better than expected
		N	*39*	*724*	*29*
	Worse than expected	*20*	19	**1**	**0**
	As expected	*760*	**20**	722	**18**
	Better than expected	*12*	**0**	**1**	11

© 2021 Panagiotou OA et al. *JAMA Network Open.*

eTable 9. Changes in outlier status by quartiles of % MA admissions for CHF

% MA Admissions	Hospital 30-Day RSRR in TM	Hospitals, N	Hospital 30-Day RSRR in MA & TM		
**<8.1%**			Worse than expected	As expected	Better than expected
		N	*37*	*891*	*32*
	Worse than expected	*28*	27	**1**	**0**
	As expected	*900*	**10**	886	**4**
	Better than expected	*32*	**0**	**4**	28
**8.1%-16.5%**			Worse than expected	As expected	Better than expected
		N	*92*	*815*	*52*
	Worse than expected	*77*	69	**8**	**0**
	As expected	*838*	**23**	804	**11**
	Better than expected	*44*	**0**	**3**	41
**16.5%-28.3%**			Worse than expected	As expected	Better than expected
		N	86	792	81
	Worse than expected	*70*	69	1	0
	As expected	*829*	17	785	27
	Better than expected	*60*	0	6	54
**≥28.3%**			Worse than expected	As expected	Better than expected
		N	*125*	*751*	*84*
	Worse than expected	*67*	61	**6**	**0**
	As expected	*850*	**64**	738	**48**
	Better than expected	*43*	**0**	**7**	36

© 2021 Panagiotou OA et al. *JAMA Network Open.*

eTable 10. Changes in outlier status by quartiles of % MA admissions for pneumonia

**% MA Admissions**	**Hospital 30-Day RSRR in TM**	**Hospitals, N**	**Hospital 30-Day RSRR in MA & TM**		
**<6.7%**			Worse than expected	As expected	Better than expected
		N	*69*	*873*	*61*
	Worse than expected	*57*	56	**1**	**0**
	As expected	*903*	**13**	872	**18**
	Better than expected	*43*	**0**	**0**	43
**6.7%-14.3%**			Worse than expected	As expected	Better than expected
		N	*109*	*819*	*66*
	Worse than expected	*97*	93	**4**	**0**
	As expected	*850*	**16**	815	**19**
	Better than expected	*47*	**0**	**0**	47
			Worse than expected	As expected	Better than expected
**14.3%-25.2%**		*N*	145	771	94
	Worse than expected	*110*	107	3	0
	As expected	*835*	38	761	36
	Better than expected	*65*	0	7	58
**≥25.2%**			Worse than expected	As expected	Better than expected
		N	*155*	*754*	*94*
	Worse than expected	*106*	99	**7**	**0**
	As expected	*833*	**56**	732	**45**
	Better than expected	*64*	**0**	**15**	49

© 2021 Panagiotou OA et al. *JAMA Network Open.*

eFigure 1. Flowchart of eligible hospitals for AMI, CHF, and pneumonia

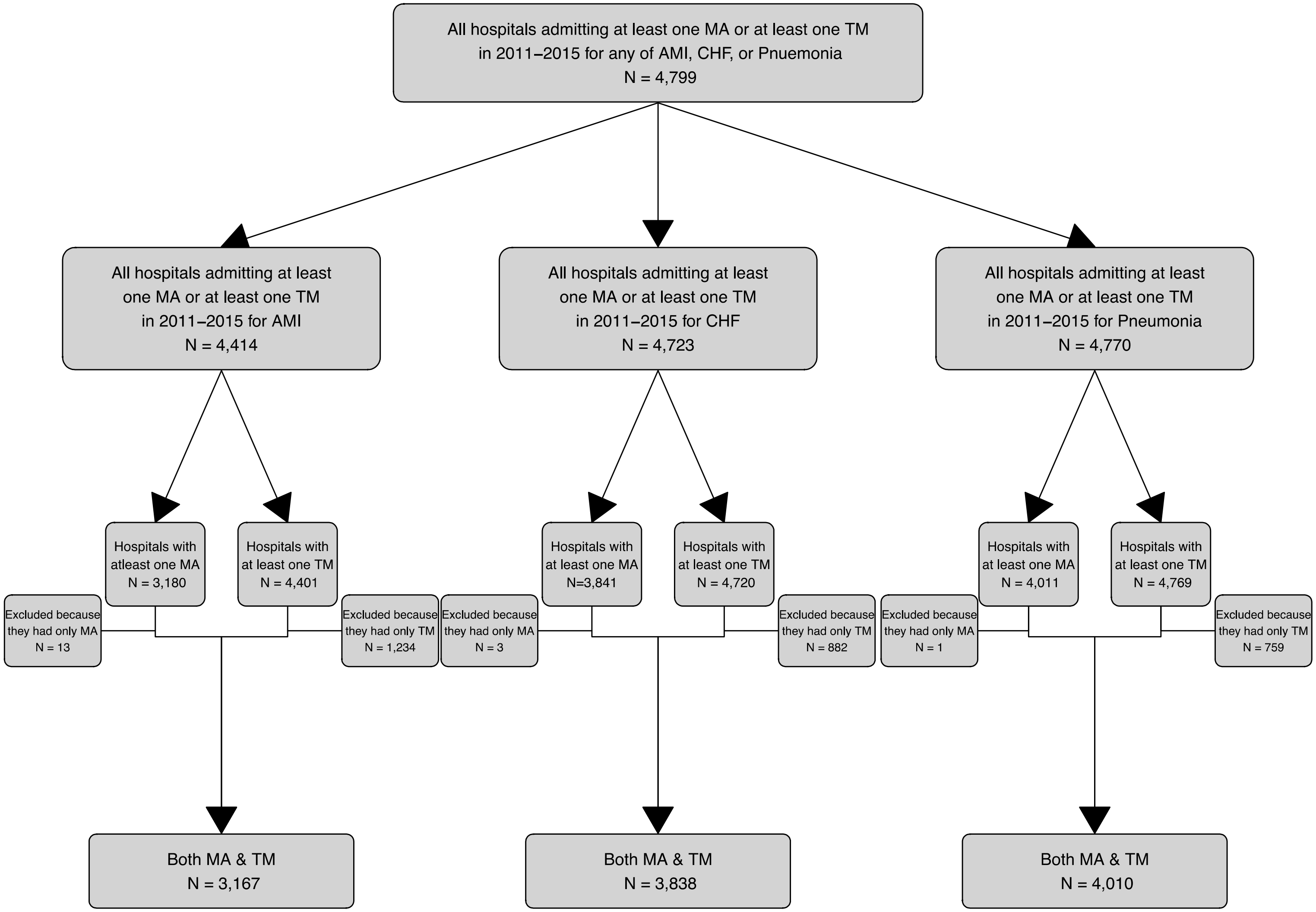

© 2021 Panagiotou OA et al. *JAMA Network Open.*

eFigure 2. Receiver operating characteristic curves for the 30-day readmission model in TM, MA and all Medicare

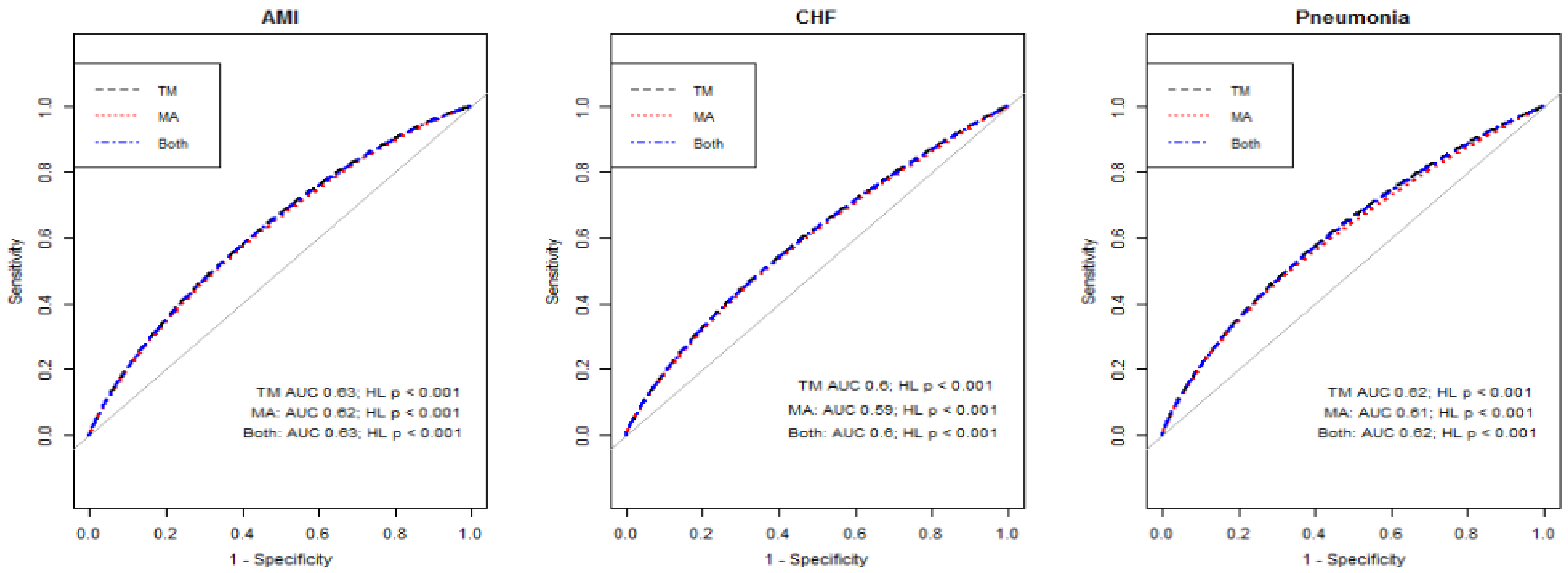

Note: RSRR's derived from TM model (coefficients) fit using only hospitals with both TM and MA.

AUC = area under the curve; HL = p-value of Hosmer-Lemeshow statistic

© 2021 Panagiotou OA et al. *JAMA Network Open.*

eFigure 3. Distribution of Hospital-Specific 30-Day Risk Standardized Readmission Rates after AMI for Traditional Medicare, Medicare Advantage, and all Enrollees

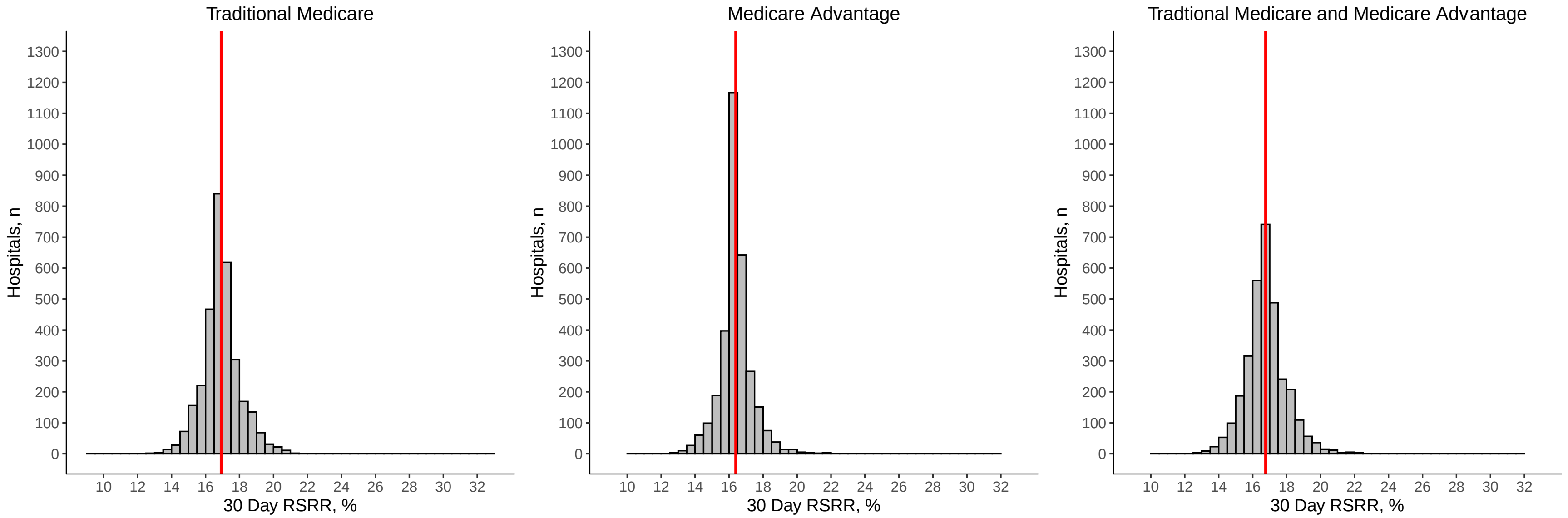

© 2021 Panagiotou OA et al. *JAMA Network Open.*

eFigure 4. Distribution of Hospital-Specific 30-Day Risk Standardized Readmission Rates after CHF for Traditional Medicare, Medicare Advantage, and all Enrollees

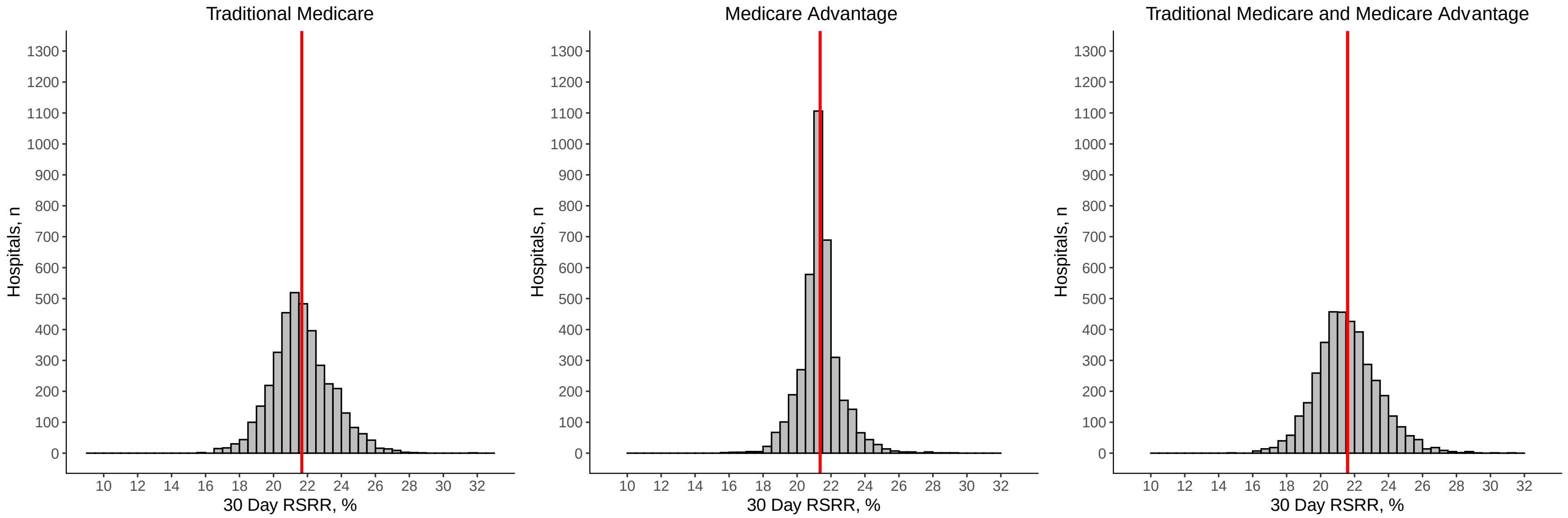

© 2021 Panagiotou OA et al. *JAMA Network Open.*

eFigure 5. Distribution of Hospital-Specific 30-Day Risk Standardized Readmission Rates after Pneumonia for Traditional Medicare, Medicare Advantage, and all Enrollees

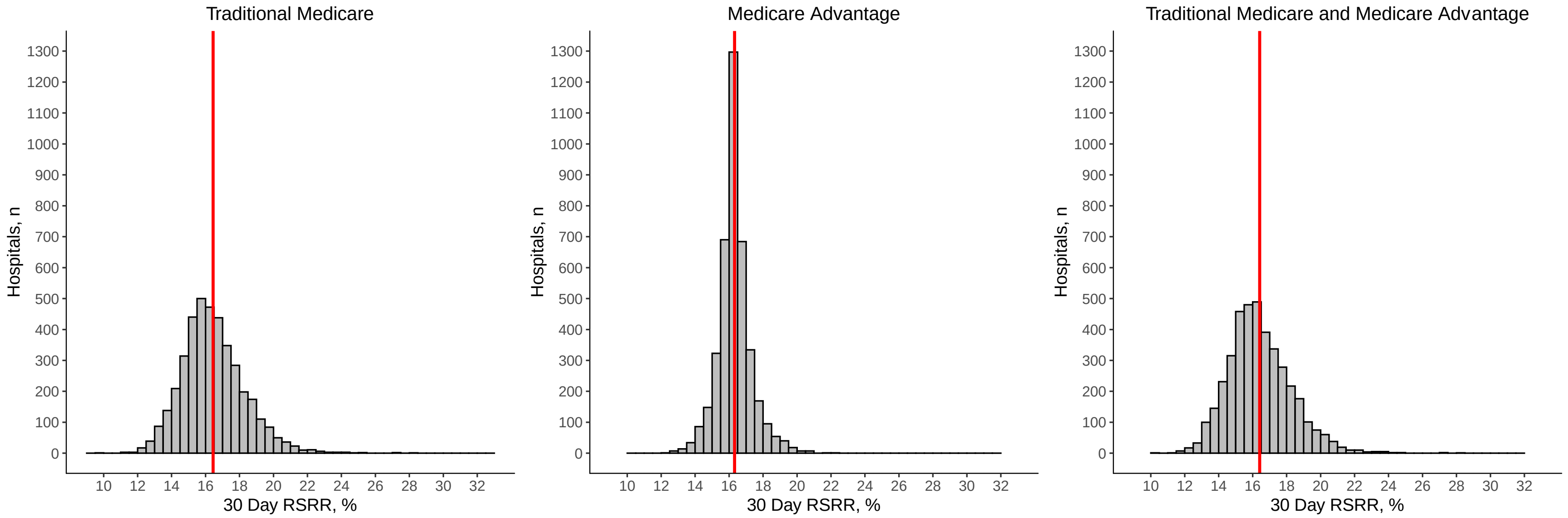

© 2021 Panagiotou OA et al. *JAMA Network Open.*

eFigure 6. Correlation between Hospital-Specific 30-Day Readmission Rates after AMI, CHF, and Pneumonia in Traditional Medicare and both Traditional Medicare and Medicare Advantage Enrollees (top); and Traditional Medicare and Medicare Advantage Enrollees (bottom)

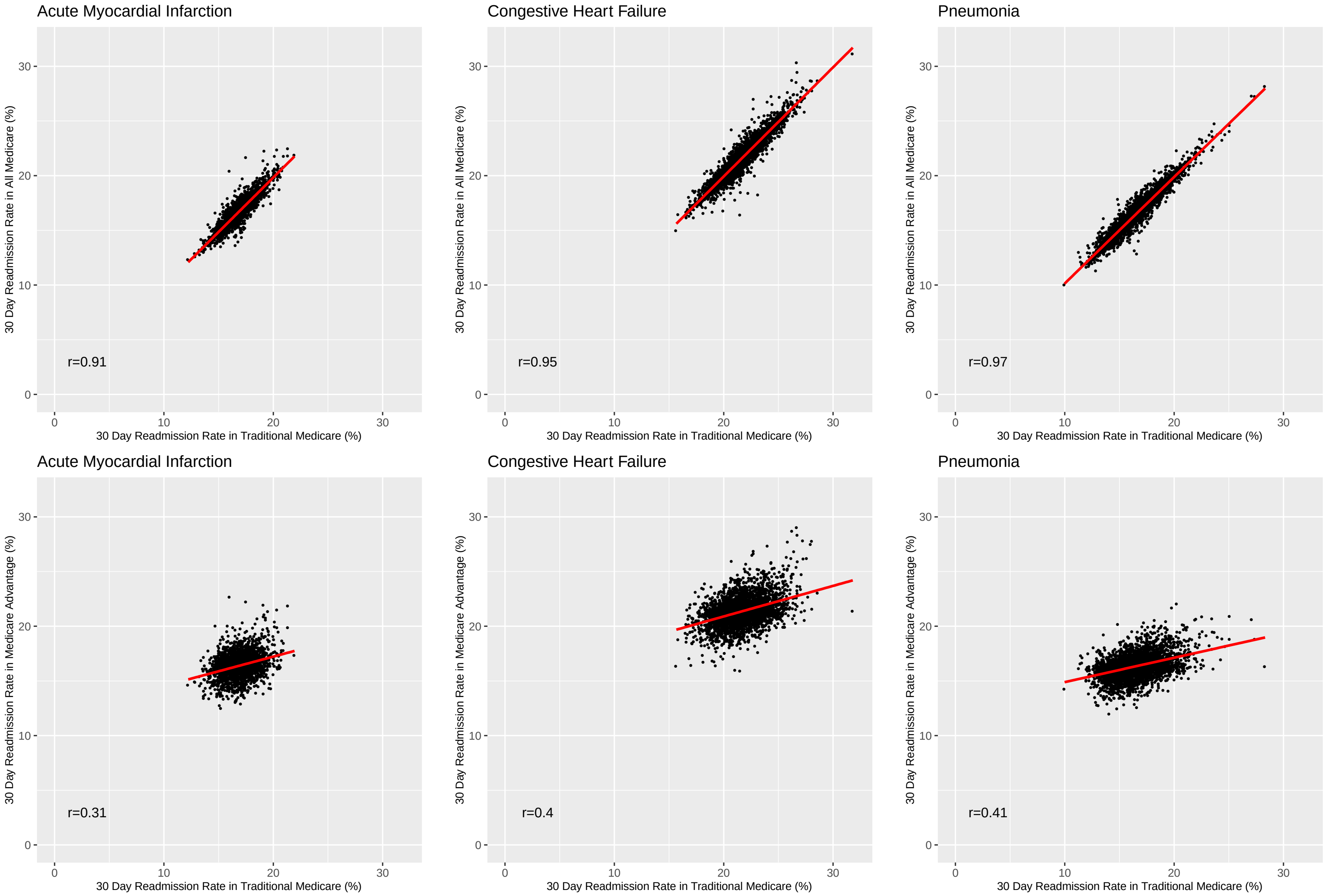

© 2021 Panagiotou OA et al. *JAMA Network Open.*

eFigure 7. Bland-Altman Plots Comparing 30-Day RSRRs for traditional Medicare Patients and All Medicare Patients

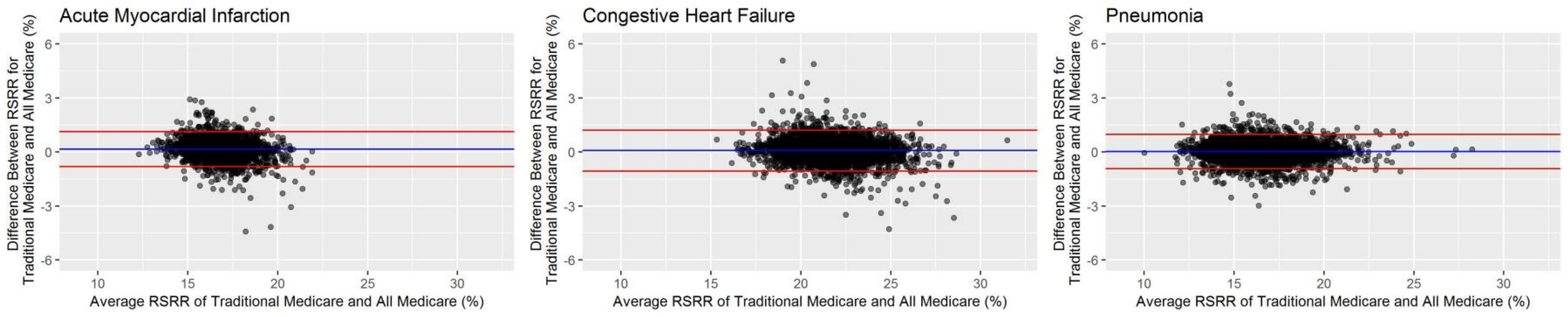

The blue line in each plot corresponds to the mean difference of the traditional Medicare-based estimation of 30-day RSRR and all Medicare-based estimation of 30-day RSRR. Horizontal red lines show the limits of agreement between the 2 measures of 30-day RSRR. We used a conventional statistical threshold of 1.96 standard deviations (SDs) as the limit of agreement between the TM-based RSRR and the RSRR based on both TM and MA. When the agreement is high, the difference between each hospital's RSRRs for its TM patients and all its Medicare patients is expected to be low and the majority of differences across all hospitals fall within the limits of agreement.

© 2021 Panagiotou OA et al. *JAMA Network Open.*

eFigure 8. Hospital performance by condition based on Traditional Medicare and all Medicare patients

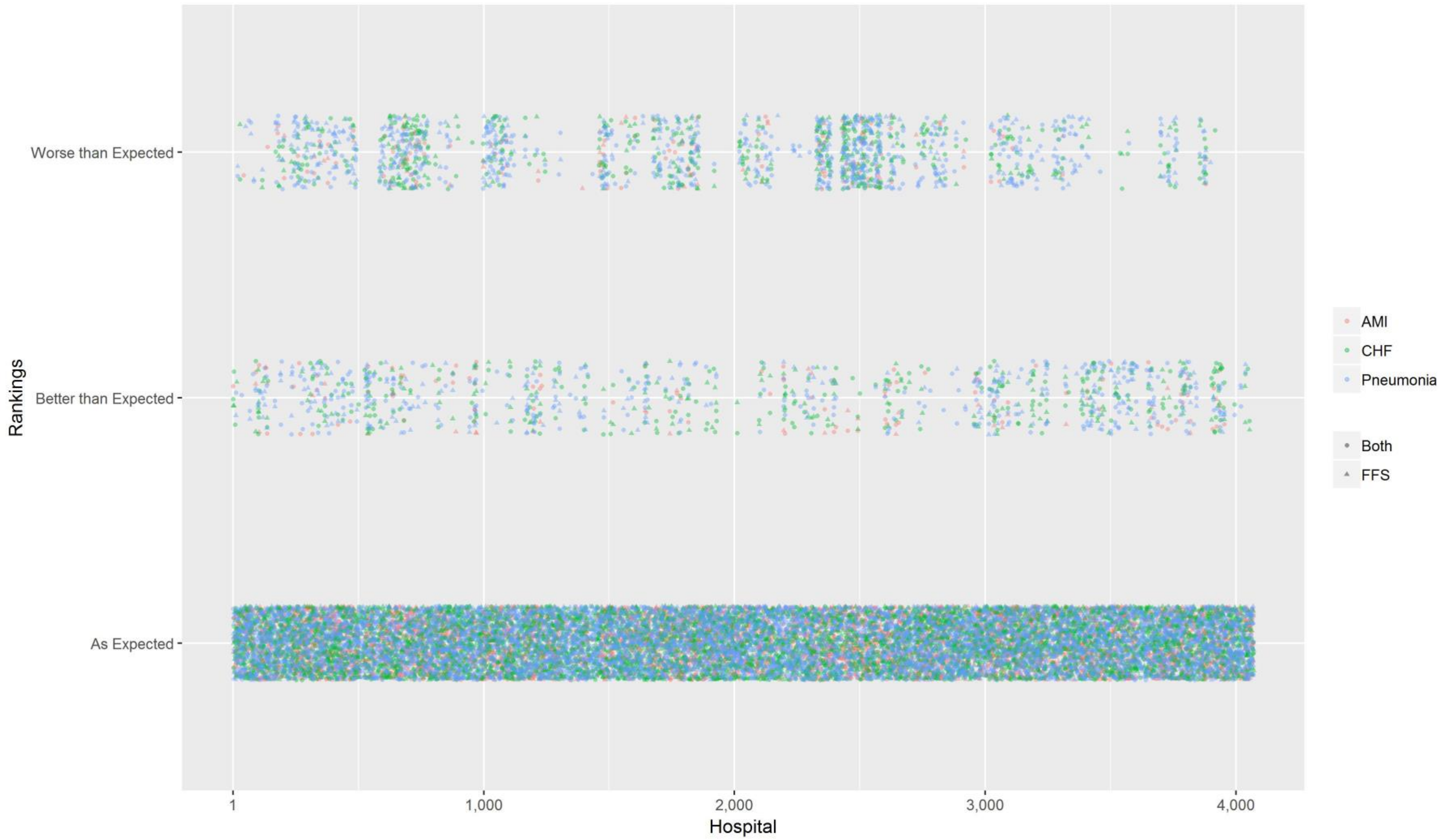

Each data point represents a single hospital and its performance (as-expected, better than expected, worse than expected) for AMI, CHF, and pneumonia based on the hospital's TM patients only and all of its Medicare patients.

There were 486 hospitals with worse-than-expected readmission rates for at least one condition according to TM data alone, and 37 (8%) of those were no longer worse-than-expected based on both TM and MA patients.

© 2021 Panagiotou OA et al. *JAMA Network Open.*
